# The importance of equally accessible genomic surveillance in the age of pandemics

**DOI:** 10.1007/s42977-023-00164-5

**Published:** 2023-05-18

**Authors:** Safia Zeghbib, Gábor Kemenesi, Ferenc Jakab

**Affiliations:** 1 National Laboratory of Virology, Szentágothai Research Centre, University of Pécs, Pecs, Hungary; 2 Faculty of Sciences, Institute of Biology, University of Pécs, Pecs, Hungary

**Keywords:** Genomic epidemiology, Sequencing, Disease containment, Pandemic, Sequencing equity

## Abstract

Genomic epidemiology is now a core component in investigating the spread of a disease during an outbreak and for future preparedness to tackle emerging zoonoses. During the last decades, several viral diseases arose and emphasized the importance of molecular epidemiology in tracking the dispersal route, supporting proper mitigation measures, and appropriate vaccine development. In this perspective article, we summarized what has been done so far in the genomic epidemiology field and what should be considered in the future. We traced back the methods and protocols employed over time for zoonotic disease response. Either to small outbreaks such as the severe acute respiratory syndrome (SARS) outbreak identified first in 2002 in Guangdong, China, or to a global pandemic like the one that we are experiencing now since 2019 when the severe acute respiratory syndrome 2 (SARS-CoV-2) virus emerged in Wuhan, China, following several pneumonia cases, and subsequently spread worldwide. We explored both the benefits and shortages encountered when relying on genomic epidemiology, and we clearly present the disadvantages of inequity in accessing these tools around the world, especially in countries with less developed economies. For effectively addressing future pandemics, it is crucial to work for better sequencing equity around the globe.

## Introduction

Genomic epidemiology permits pathogen transmission tracing through all spatial scales from local outbreaks to worldwide pandemics; therefore, it is a powerful tool for outbreak management and intervention fine-tuning. On the other hand, merging personal metadata with genomic data may highlight demographic factors triggering transmission patterns. Thus, molecular epidemiology progressed toward being a crucial element of outbreak response (Gardy and Loman 2018; Hill et al. 2021). For instance, the severe acute respiratory syndrome coronavirus (SARS-CoV) outbreak which occurred in 2003 in China was characterized first by direct sequencing of clinical samples of index cases. Both molecular analysis and epidemiological data demonstrated multiple introductions of the virus and helped in establishing diagnostic tests and the implementation of mitigation measures for disease containment (Cherry and Krogstad 2004; Ruan et al. 2003). Between 2013 and 2016, the potential of genomic epidemiology to frame an outbreak response became more tangible during the West African Ebola outbreak (Holmes et al. 2016). This outbreak was the starting point for large-scale real-time molecular epidemiology; herein, both advancements in high-throughput next-generation sequencing (NGS) and the development of portable sequencers which can be deployed in the field played a key role in data generation within days or hours from the sampling time. Using these data, the origin of the outbreak was determined as a single spillover event followed by constant human-to-human transmission. In addition, to understand transmission patterns, the possibility of viral persistence in immune-privileged sites such as testes and sexual transmissibility of the disease from the outbreak survivors was proven via molecular epidemiology sequencing tools as well. It framed recommendations for male survivors as they have to take repeated semen samples after recovery to test for viral RNA presence (Mate et al. 2015; Subissi et al. 2018).

In late 2019, the emergence of a novel coronavirus, namely, SARS-CoV-2 in Wuhan, China, later responsible for a global pandemic declared in March 2020 by the World Health Organization has once again proved the central role of molecular epidemiology and data sharing in the pandemic response. First genomes were obtained on January 3, 2020, from bronchoalveolar lavage fluid samples by combining Illumina sequencing, Sanger sequencing, and Nanopore sequencing, and sequences were directly uploaded to the GISAID database (Elbe and Buckland‐Merrett 2017; Tan et al. 2020). Based on this genomic information, an RT-PCR diagnostic test was rapidly developed, and the diagnostic protocol was communicated by the WHO (Corman et al. 2020). Predictably, several control measures such as social distancing, the use of face masks, and isolation were executed to limit disease transmission; however, this was not sufficient to hamper the spread of COVID-19 resulting in the need for vaccine development. Once again genomic data sharing turned out to be crucial and 66 days after the first viral sequence was released the phase 1 clinical trial of a novel vaccine technology based on the mRNA of the coronavirus spike protein already started (Corbett et al. 2020; Dong et al. 2021). Moreover, the intensive sequencing and data sharing helped to shed the light on emerging variants worldwide and, thereafter, characterize their severity, transmissibility, and immune evasion, thus helping mitigation measure implementation, reconsidering the treatment protocol, and raising awareness regarding the importance of vaccination (Andreata-Santos et al. 2022; Takeshita et al. 2022). For instance, in the autumn of 2020, a surge in COVID-19 cases was observed in the UK as well as S-gene target failures (SGTF) in community-based diagnostic PCR tests. Following sequencing, several non-synonymous amino acid replacements in the spike protein later confirmed to be associated with both increased infectivity and severity were characterized. The N501Y replacement was linked to an elevated binding activity to the ACE2 receptor, the P618H approximating the furin cleavage site, and the deletion at positions 69 and 70 of the spike protein (Δ69-70) which was related to diagnostic test failure for the widely used Thermo Fisher TaqPath probe (Bonnet et al. 2022). Subsequently, the infectivity and the severity of the SARS-CoV-2 Alpha variant were deduced based either on epidemiological data or experimental data. By way of illustration, the risk of transmission for the Alpha variant communicated based on 15 studies was 45–71% higher. Likewise, hospitalization increased by 1.7-fold whereas admission to the intensive care unit (ICU) was 2.3 times higher when compared to the wild type. However, at that time, the Alpha variant had less impact on the vaccine, and the vaccine was still protective (Lin et al. 2021a, b). The continuous molecular tracking confirmed its strength by unveiling the emergence of new variants while the epidemiological data highlighted some disease tendencies and guided the experimental investigation. Based on genomic epidemiology and the WHO reports (as of July 19, 2022), we have five variants of concern namely Alpha, Beta, Gamma, Delta, and Omicron of which only the Omicron variant remains, and the others were de-escalated (Carabelli et al. 2023). Luckily, Omicron was reported to be less severe than previous variants especially Delta; nevertheless, it evolved to effectively escape both infection- and vaccine-induced immunities, especially with the appearance of several sub-variants (Ao et al. 2022; Nealon and Cowling 2022). This changed the vaccination strategy, by way of illustration Israel decided to give a fourth vaccine booster dose to the most vulnerable persons as a way to prevent severe disease; however, this dose brings back the antibodies levels to those observed after the third dose but then again it provides only a modest boost (Bar-On et al. 2022; Mallapaty 2022). Subsequently, updated vaccination strategies targeting the novel Omicron variants are needed; herein, Moderna developed a bivalent mRNA-based vaccine targeting both the Wuhan strain and the Omicron variant which is already in phase 2/3 trial (Chalkias et al. 2022). Rapid sequencing of emerging variants is crucial to effectively face epidemiological challenges and to support vaccine development efforts by rapidly providing high-quality genomic data. Sequencing and sequence data sharing are now the main technological supplementation of modern, mRNA-based vaccine development. The developed second-generation COVID-19 vaccine produced a superior titer of neutralizing antibodies against the Omicron variant without a decrease in the original Wuhan Hu1 strain + D614G mutation neutralizing antibodies (Chalkias et al. 2022). As we all know, globalization and travel played a crucial role in SARS-CoV-2 disease dissemination, which is neither the first pandemic nor the last; however, it illustrated perfectly how our society is unprepared to face zoonotic diseases (Arora et al. 2021; Machado et al. 2021). Modern sequencing technologies are applicable for other viruses with bigger genome size as well. The current Monkeypox virus outbreak in the Western Hemisphere is highly supported and monitored by genomic epidemiology toolkits. In May 2022, a British citizen who had traveled to Nigeria where the virus is endemic was confirmed positive. Thereafter, other unrelated cases were reported from at least 47 countries in five continents. Phylogenetic analysis indicated that all sequences originated from the West African (WA) clade without excluding the hypothesis of multiple introductions from a single origin and the occurrence of super spreader events probably leading to the rapid global dissemination (Vivancos et al. 2022). So far, both the origin and the evolutionary trajectory of the novel outbreak are under investigation; however, we noticed the importance of a generalized and correlated molecular tracking system which has to involve poor and low-income countries considering that they might be central in the transmission loop (Gigante et al. 2022; Isidro et al. 2022).

### Tracing the evolution with sequencing

Although the mutation occurrence in viruses is expected with higher frequencies in RNA viral families, the SARS-CoV-2 pandemic demonstrated that the rise of novel variants is multifactorial and not fully understood. Furthermore, predicting the evolutionary trend of the virus to hamper the emergence of novel lineages turned out to be extremely hard and complex (Peck and Lauring 2018). Indeed, the viral RNA-dependent RNA polymerase low replication fidelity is a key factor in mutation incidence; however, prior studies conducted during the current pandemic demonstrated the role of host RNA editing machineries such as the apolipoprotein B mRNA editing catalytic polypeptide-like proteins (APOBEC) and adenosine deaminase acting on RNA proteins (ADAR) in generating mutations (Mourier et al. 2021). Additionally, the selective pressure exercised during antiviral drug treatments, monoclonal antibody use, or convalescent plasma transfusion might favor the appearance of immune escape mutations. Likewise, the extraordinary intra-host environment of immunocompromised patients favoring chronic infections should not be neglected in the emergence of immune escape mutations (Salehi-Vaziri et al. 2022; Sonnleitner et al. 2022). All this needs thorough real-time monitoring. A lot of efforts are deployed to effectively perform mutation tracing using genomic epidemiology.

So far, we noticed two types of variants; worldwide dominants such as Delta and Omicron and lineages that triggered local surges but did not reach global dominance such as Gamma and Mu (Chavda et al. 2022). For instance, the Delta variant initially detected in India on May 7, 2021, was declared as a variant of concern by the WHO as it held three characteristic mutations in its spike protein facilitating immune evasion, resistance to monoclonal antibodies, and increased transmissibility. Despite the implementation of travel restrictions in order to hinder its spread, as of July 27, 2021, the Delta variant reached 132 countries and subsequently overcame the British Alpha variant. This lineage was associated with a surge in COVID cases worldwide with an increased hospitalization, ICU admissions, and a higher mortality rate. Similarly, the intensive real-time sequencing unveiled the emergence of a novel Omicron second-generation variant, namely, BA.2.75 with over 80 mutations with more than nine in the spike protein including the two key replacements G446S and R493Q which increase significantly the immune escape and the attachment to the ACE2 host cell receptors (Chakraborty et al. 2022). It was detected first in India, and then, it spreads to other countries including Australia, the UK, the US, Israel, Japan, Germany, Canada, and New Zealand, once again concerns are raised despite that the WHO is yet to officially flag it as a variant of concern (Peacock 2022). This demonstrates the importance of continuous tracking for spotting variants right in time and taking one step ahead to better control and limit repercussions. Nevertheless, a key observation during the COVID pandemic is the repetition of the same scenario at each variant emergence, while some countries have both diagnostic and sequencing capacities and are able to perform a continuous surveillance with real-time data reports, other countries, especially low- and middle-income countries, have a limited access to molecular diagnostics and sequencing resulting in a patchy surveillance with a delayed data reports and underrepresentation of the real cases (Chen et al. 2022).

**Fig. 1:**
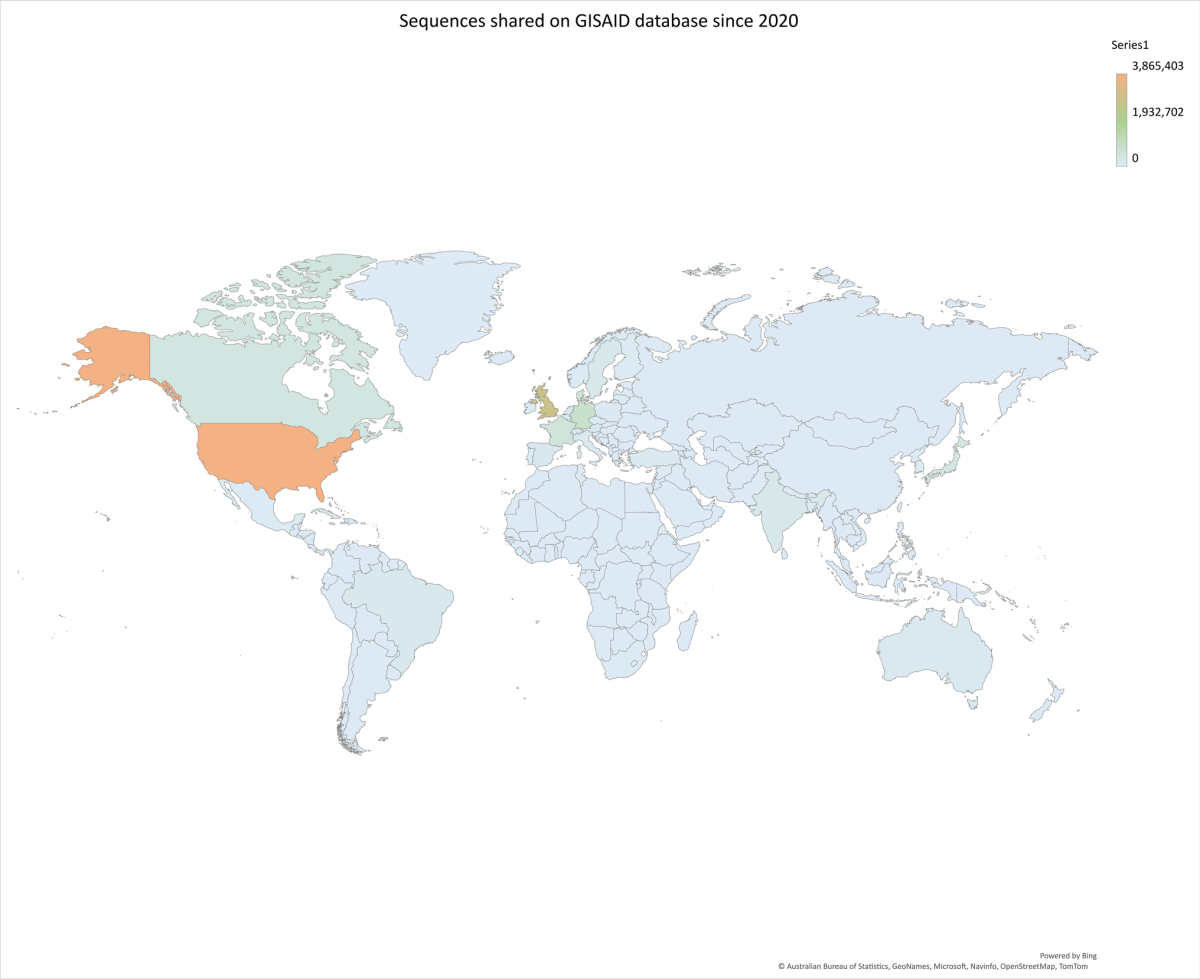
The inequities of genomic surveillance throughout the world, based on the submitted SARS-CoV-2 complete genomic sequences to the GISAID database (accessed: 2022.08.17.). The map indicates well-represented and less-represented regions in the world based on the total number of sequences uploaded to the GISAID database

To illustrate this, as of July 6, 2022, the GISAID database englobed 2,838,148 genomes originated in the United Kingdom, with a time lapse of maximum 10 days between the sampling time and the data sharing. Whereas only 317 Algerian sequences were uploaded to the database of which some were sequenced 10 months after sampling (Elbe and Buckland‐Merrett 2017). Similarly, the database has only 58 sequences from Chad which were sequenced by the National Institute for Biomedical Research in the Democratic Republic of Congo (Fig. 1). Note that in poor countries, where the vaccination is not conducted, there is a high probability for novel variant emergence; however, the lack of real-time genomic surveillance and the limited resources result in novel variants going unnoticed. Yet again, this highlights the importance of generalized worldwide genomic surveillance to reflect or at least to get closer to the real-life scenario of the pandemic.

#### The methodology used to track the SARS-CoV-2 pandemic

Advances in SARS-CoV-2 sequencing have permitted the identification of new emerging variants and the monitoring of their epidemiology, evolution, and spread. As the pandemic progressed, extensive efforts were deployed by the scientific community to standardize sequencing protocols and bioinformatic pipelines in order to get accurate consensus genomes. The SARS-CoV-2 genome was first unveiled by Chinese scientists using next-generation sequencing platform (Illumina) and applying paired-end deep meta-transcriptomic sequencing protocol (Wu et al. 2020). However, at that time, the pathogen was unknown, and no reference genomes were available. Besides, not all countries have the experience or access to NGS platforms which are usually imposing and require several preparation steps and high computational power. The MinION portable sequencer commercialized by Oxford Nanopore Technology (ONT) characterized by a minimal laboratory environment requirement and a reduced time for preprocessing of the sample compared to NGS platforms offered advantageous opportunities, especially for middle- and low-income countries with limited sequencing experience (Hourdel et al. 2020). Early in the pandemic, an amplicon-based sequencing protocol was adopted. It is founded on first-strand cDNA synthesis followed by genome amplification using tiled multiplexed primers to produce amplicons covering the whole genome. It was standardized for both Illumina and MinION sequencing platforms. Since its initial release, the ARTIC SARS-CoV-2 sequencing protocol became one of the most regular reference methods for several laboratories around the world and now different commercial kits are also accessible (Quick et al. 2017; Rosenthal et al. 2022). We implemented the same method for genomic surveillance. The importance of such surveillance in variant monitoring is well highlighted in numerous papers. During our investigation period, we could detect an 84-bp deletion in the spike gene using the same sequencing methods, and we performed an end-point PCR and Sanger sequencing for confirmation (Kemenesi et al. 2020, 2021). This deleterious mutant was described in a patient with coinfection by two variants. However, the increasing number of mutations in the new emerging variants represents a probable challenge for the amplicon-based method, since the mutated primers may potentially trigger amplicon dropout or patchy sequencing coverage; therefore, it needs continuous validation and fine-tuning. To tackle this, Arana and colleagues developed a sequencing method that combines both long amplicon-based reads and short amplicon-based reads to improve genomic coverage and effectively capture SNPs, deletions, or insertions within the genome (Arana et al. 2022). The hybrid genome assembly method is by way far the most effective sequencing method; nevertheless, it is more expensive and more time-consuming. In parallel, direct RNA sequencing from native swab samples was also performed, this relies on direct sequencing of RNA without a cDNA stage; hence, the native RNA characteristics might be explored without the contamination artifacts caused by the cDNA amplification, this is consequential to study mutations and viral transcriptomics; however, it has some limitations such as the amount of the input RNA and its quality (Davidson et al. 2020; Vacca et al. 2022).

To perform an efficient genomic epidemiology investigation during the pandemic, several analytic methods were used to examine the resultant genomes from the sequencing step. By way of illustration, in the first SARS-CoV-2 pandemic wave between March 2020 and April 2020, we performed a phylogenetic analysis to visualize the clustering of our newly sequenced genomes. The dataset comprised 32 Hungarian genomes in addition to 105 sequences sampled worldwide. The analysis supported the multiple introductions origin of the disease. Similarly, the large-scale haplotype network analysis of 7864 high-quality sequences retrieved from the GISAID database in addition to our 32 Hungarian genomes supported the multiple introductions theory and demonstrated some local transmissions (Kemenesi et al. 2020). On the other hand, later in the pandemic, we collaborated with Pasteur Algérie, and we analyzed sequences from the first and second waves, the dataset involved 29 Algerian genomes and 66 sequences sampled worldwide. We performed a time-dated phylogenetic analysis to estimate the starting date of the Algerian pandemic which correlated with the reported epidemiological data. Furthermore, we performed a phylogeographic analysis to illustrate the spreading route within the country. As a result, we could emphasize the importance of both international and local travel in disease spread, and we highlighted the missing unsampled data. In a similar manner, we characterized the mutational pattern on both nucleotide and amino acid levels, also we could perform molecular tracing based on amino acid substitutions, and we predicted the effect of the substitutions on the corresponding proteins. Finally, we assessed the selective pressure among the protein-coding genes, which were overall evolving under a negative selection (Zeghbib et al. 2021).

Another example of data analysis for the purpose of genomic epidemiology is the phylodynamic which specifies some features of viral demography such as viral population growth and decline and its structure. Basically, it studies the evolution of a viral population based on its genomic sequences. It also permits the direct estimation of the basic reproductive number (R0) and the effective reproductive number (Re) from the sequence data (Cardona-Ospina et al. 2021). With the emergence of new variants, another important tool that is directly linked to infectivity and immune escape evaluation is 3D protein modeling. Mutations occurring in the receptor-binding domain (RBD) of the spike protein play a key role in the infection process by interacting with ACE2 cell receptors. Hence, it is important to develop prediction tools to assess the affinity of novel RBD variants to ACE2 receptors in order to take a step ahead and prepare for better response measures, therapies, and vaccination strategies. *In silico* analysis is crucial to step from pandemic response to pandemic prediction and preparedness (Tragni et al. 2022).

#### Pros, cons, and future perspectives

Over the last decade, genomic epidemiology has established itself as a tool with a powerful potential to both tracks and respond to outbreaks. As a key use, it permitted tracking back the origin of the disease, when the SARS-CoV outbreak occurred in China in 2003, the civet cat was supposedly the direct origin of the virus; however, the genomic studies demonstrated that the civet cat is just an intermediate host, whereas the natural reservoir hosts are bats (Shi and Hu 2008). Furthermore, in order to develop better therapeutic strategies, meta-transcriptomic analysis permitted the revelation of enhanced pathways implicated in the SARS-CoV-2 pathogenesis (Alberts et al. 2022). On the other hand, disease transmission occurs at different times and spaces and depends on the type of pathogen, immunity, host movement pattern, population density, and other factors. Molecular epidemiology allows the discovery of how pathogens are related over space and time from small outbreaks to worldwide pandemics. Hence, the inclusion of metadata such as the sampling location and sampling date is of a great importance for such investigations (Hill et al. 2021). Above all, genomic epidemiology permitted the development of precise diagnostic tools which might be modified in accordance with the genomic sequences (Rabaan et al. 2020). It allowed the establishment of effective mitigation measures such as travel bans and border closures to hamper the disease spread (Ari et al. 2022). And importantly, it played a role in vaccine and drug development with adjustment possibility following novel variant emergence (Ladner et al. 2019). Nonetheless, the current pandemic response had several, not neglectable shortcomings. In middle- and low-income countries, there is a lack of sequencing infrastructures or even molecular-based practices. For instance in Algeria which is the largest African country with roughly 45 million inhabitants, only one sequencing center is available, the access to PCR tests is also inadequate to monitor virus spread. On the other hand, researchers in many developing countries do not have the technologies, resources, and capacities to fully perform genomic-related research. Hence, collaboration establishment with more developed countries and grant applications might be a huge step toward building capacities and getting the required knowledge and training to perform efficient genomic epidemiology (Ladner et al. 2019). Not to mention that the COVID-19 pandemic emphasized the crucial role played by international organizations such as the World Health Organization (WHO) in facilitating worldwide laboratory networking to support equitable access to whole genome sequencing and genomic surveillance ( Lin et al. 2021a, b). For instance, at the beginning of the pandemic, Algeria did not have any established sequencing facility at Pasteur institute; therefore, since it is a part of the WHO reference laboratory, the samples were shipped to Pasteur France for sequencing and genomic analysis purposes, hence generating the first data regarding the Algerian pandemic (Gámbaro et al. 2020). Besides, another aspect of the pandemic remains poorly investigated; the reverse zoonotic part which is crucial to get a perspective regarding the virus evolution and adaptation as it might favor the rise of new variants (Goraichuk et al. 2021). Therefore, the establishment of a robust One Health-based investigation is essential, this approach integrates professionals from different disciplines such as human medicine, veterinary medicine, and ecology, to improve both humans and animals (Ghai et al. 2022). In addition, since the emergence of infectious diseases is a worldwide problem, especially with activities such as travel and trade which facilitate the movement of the virus with the potential threat of a novel pandemic. The implementation of worldwide generalized surveillance is a must for a pandemic response as well as future pandemic prediction and preparedness (Coloma and Harris 2009). For effectively addressing future pandemics, it is crucial to work for better genomic sequencing equity around the globe with a targeted funding strategy, more efficient distribution of cheap and compact sequencing technologies, better networking and knowledge transfer, and better policy decision-making. For instance, to minimize the considerable investment in whole genome sequencing and computing infrastructures, the adequate choice of the sequencing and data storage platforms (cloud-based or onsite) must be evaluated against the internet reliability, confidentiality risk, cost, and easiness of data retrieval (Inzaule et al. 2021). Whereas, to reduce the per-sample cost multiplexing samples with barcodes proved to be profitable. On the other hand, employing a skilled workforce is critical as a wide range of experts is needed to effectively implement genomic surveillance ranging from molecular biologists for data generation to bioinformaticians and molecular epidemiologists to analyze and interpret the data. Furthermore, local experts in equipment installation and maintenance are needed to avoid both delays and additional costs from external outsourcing (Inzaule et al. 2021). Likewise, leadership and effective coordination are critical to establishing a genomic surveillance network relying on shared resources and joint efforts for successful cross-border disease control (Lee et al. 2020). Additionally, the use and adaptation of already established surveillance systems in the frame of pandemic preparedness for other respiratory viruses like the influenza virus are of a great benefit for the current pandemic, it helps to quickly operationalize COVID-19 strategic response plans to face the current pandemic (Marcenac et al. 2022). Importantly, global efforts must be deployed to improve genomic surveillance and establish sustainable research fundings for strengthening pathogen surveillance at the human, animal, and human–animal interfaces (Brito et al. 2022).
